# Diagnostic delay is common among patients with hypophosphatasia: initial findings from a longitudinal, prospective, global registry

**DOI:** 10.1186/s12891-019-2420-8

**Published:** 2019-02-14

**Authors:** Wolfgang Högler, Craig Langman, Hugo Gomes da Silva, Shona Fang, Agnès Linglart, Keiichi Ozono, Anna Petryk, Cheryl Rockman-Greenberg, Lothar Seefried, Priya S. Kishnani

**Affiliations:** 1 Department of Pediatrics and Adolescent Medicine, Johannes Kepler University Linz, Linz, Austria; 2 Feinberg School of Medicine, Northwestern University and Lurie Children’s Hospital, Chicago, IL USA; 3 Alexion Pharmaceuticals, Inc., Boston, MA USA; 4 APHP, Bicêtre Paris-Sud, University Paris Sud, Paris-Saclay, Le Kremlin Bicêtre, Paris, France; 5 Department of Pediatrics, Osaka University, Graduate School of Medicine, Suita, Osaka Japan; 6 Rady Faculty of Health Sciences, Max Rady College of Medicine, and Children’s Hospital Research Institute of Manitoba, University of Manitoba, Winnipeg, Manitoba Canada; 7 Orthopaedic Clinic, König-Ludwig-Haus, University of Würzburg, Würzburg, Germany; 8 Department of Pediatrics, Duke University Medical Center, 2301 Erwin Rd, Durham, NC 27710 USA

**Keywords:** Hypophosphatasia, Natural history, Rare diseases, Alkaline phosphatase, Asfotase alfa

## Abstract

**Background:**

Hypophosphatasia (HPP) is a rare, systemic disease caused by mutation(s) within the *ALPL* gene encoding tissue-nonspecific alkaline phosphatase (ALP). HPP has a heterogeneous presentation, which coupled with its rarity, often leads to missed/delayed diagnosis and an incomplete understanding of its natural history. To better understand the epidemiology and clinical course of HPP, including timing of diagnosis after first reported manifestation, we present baseline data for patients enrolled in the Global HPP Registry.

**Methods:**

Data were analyzed from patients with an HPP diagnosis confirmed by low serum ALP activity and/or an *ALPL* pathogenic variant, regardless of prior or current treatment, according to age at enrollment (children: < 18 y; adult: ≥18 y). All analyses were descriptive.

**Results:**

Of 269 patients from 11 countries enrolled January 2015–September 2017, 121 (45.0%) were children and 148 (55.0%) were adults. The majority of children and adults were female (61.2 and 73.0%, respectively) and white (57.7 and 90.0%, respectively). Children had a median (min, max) age at earliest reported HPP manifestation of 7.2 months (− 2.3 mo, 16.0 y), which was > 12 months before diagnosis at age 20.4 months (− 0.2 mo, 16.0 y). In adults, the earliest reported manifestation occurred at a median (min, max) age of 37.6 years (0.2 y, 75.2 y), which preceded age at diagnosis (47.5 years [0.2 y, 75.2 y]) by ~ 10 years. Premature loss of deciduous teeth (48.2%, age ≥ 6 mo), bone deformity (32.5%), and failure to thrive (26.7%) were most commonly reported in the HPP-related disease history of children. Pain (74.5%), orthopedic procedures and therapies (44.6%), and recurrent and poorly healing fractures (36.5%) were most commonly reported in the HPP-related disease history of adults.

**Conclusions:**

The Global HPP Registry represents the largest observational study of patients with HPP, capturing real world data. This analysis shows that diagnostic delay is common, reflecting limited awareness of HPP, and that HPP is associated with systemic manifestations across all ages. Many patients diagnosed in adulthood had HPP manifestations in childhood, highlighting the importance of taking thorough medical histories to ensure timely diagnosis.

**Trial registration:**

Clinicaltrials.gov: NCT02306720, December 2014; ENCePP.eu: EUPAS13526, May 2016 (retrospectively registered).

## Background

Hypophosphatasia (HPP) is a rare, inherited, systemic, metabolic disease caused by genetically determined low tissue-nonspecific alkaline phosphatase (TNSALP) activity [1–4]. Low TNSALP activity leads to the extracellular accumulation of TNSALP substrates (e.g., inorganic pyrophosphate [PPi], pyridoxal 5′-phosphate [PLP]) [5, 6], resulting in both bone mineralization defects and systemic complications [3, 7]. Because of the rarity of HPP, the incidence and prevalence of this disease are difficult to estimate [3]. Commonly cited rates in the literature include an incidence of 1:100,000 in Toronto based on the local birth rate for Ontario, Canada [8], and a prevalence of 1:300,000 for perinatal/infantile HPP in France based on molecular diagnoses made from 2000 to 2009 [9]. Additional estimates include a 1:2500 prevalance of infantile HPP among the Mennonite population in Manitoba, Canada, based on live births [10], an incidence of 1:300,000–500,000 for fatal perinatal HPP in Japan [11], and a 1:900,000 prevalance of a founder mutation (c.1559delT) in Japan based on genetic analysis [12].

The clinical expression of HPP is heterogeneous, even among individuals with the same genotype or in the same family [2, 13]. Certain signs, symptoms, or complications of HPP may be more common based on the patient’s age [2, 3]. The characteristic manifestations of HPP in infants may include failure to thrive, rickets-like chest deformity, pulmonary insufficiency, craniosynostosis, and vitamin B6-responsive seizures; in toddlers, young children, and adolescents, premature tooth loss, bone deformities and rachitic-changes in long bones, and delayed motor development manifest; and, in adults, musculoskeletal pain, chondrocalcinosis (pseudogout), recurrent fractures, poorly healing fractures, or pseudofractures are characteristic [2, 3, 8]. The natural history of HPP is poorly understood at this time, likely because of the rarity and wide heterogeneity of disease presentation. Further, real world data are lacking on the disease burden for patients with HPP and, in particular, on the impact of the disease on physical function and quality of life.

Until 2015, clinical management of HPP relied mainly on supportive measures that managed the symptoms of the disease (e.g., respiratory support, orthopedic intervention, pain relief medication) but not the underlying pathophysiology [2, 3, 14, 15]. In 2015, asfotase alfa (Strensiq®, Alexion Pharmaceuticals, Inc., Boston, MA, USA), a human recombinant TNSALP enzyme replacement therapy, was approved for the treatment of patients with HPP [16–18].

The Global HPP Registry was established to improve the understanding of the disease and its impact on patients with HPP. In addition, the Registry collects data on the effectiveness and safety and tolerability of treatment with asfotase alfa as part of a postmarketing commitment to monitor real world safety and use of asfotase alfa. Data on HPP history, clinical course, signs/symptoms/complications, and burden of disease are collected from patients of all ages diagnosed with HPP, regardless of treatment status. This report presents baseline characteristics and medical history of patients enrolled in the Registry.

## Methods

The Global HPP Registry is an observational, prospective, multinational study (NCT02306720; EUPAS13526), with medical history data collected based on patient or parent/guardian recall. The Registry is sponsored by Alexion Pharmaceuticals, Inc. (Boston, MA, USA), and is overseen by a scientific advisory board comprising HPP clinical experts, including employees of Alexion Pharmaceuticals, Inc. The study protocol was approved by the institutional review board (or local equivalent) of participating study sites (see Acknowledgments section) and is being conducted in accordance with International Conference on Harmonisation Good Clinical Practice Guidelines and the Declaration of Helsinki. Before participating, all patients and/or their parent/legal guardians provided written informed consent and approval to release medical records.

### Patients

Patients of all ages who have a confirmed diagnosis of HPP are eligible for participation in the HPP Registry, regardless of asfotase alfa treatment status. Patients who were deceased before enrollment are not included in the Registry. Patients who had previously participated in an Alexion-sponsored study of asfotase alfa are allowed to enroll. Only patients who had a diagnosis of HPP confirmed by low serum ALP activity for age and sex at any time (but must have been before treatment initiation) and/or an *ALPL* pathogenic variant were included in this analysis. At minimum, patients must have also had data available for each of the following parameters: asfotase alfa treatment status; date of informed consent; date of birth (or age at enrollment in countries that did not permit collection of birthdate); and sex.

### Data collection and handling

At the time of enrollment, baseline clinical data and information related to HPP disease history are collected. Pretreatment data on HPP disease history are also collected for those who initiated treatment with asfotase alfa before being enrolled in the Registry. Investigators review patients’ medical records and submit the data to the sponsor using a secure electronic case report form (eCRF). Race and ethnicity were categorized per US National Institutes of Health recommendations.

The Registry aims to obtain data under conditions of routine clinical care in a real world setting; therefore, some patients may have missing values for some variables given that clinical care practices differ throughout the world. For purposes of data completeness, data queries were generated and distributed to sites for them to address.

### Statistical methods

We report baseline characteristics and medical history by age group at enrollment (children: age < 18 y; adults: age ≥18 y). All analyses are descriptive. Continuous variables are described using median (min, max) and mean (standard deviation [SD]), as appropriate. Categorical variables are described using frequencies and percentages. Percentages were based on the available data at the time of the analysis, which vary from variable to variable given the observational nature of the HPP Registry.

## Results

### Demographics

A total of 269 patients from 65 sites in 11 countries (Table 1) were enrolled in the HPP Registry from January 2015 through September 2017 and met the criteria for this analysis. Of these, 121 patients (45.0%) were children (median [min, max] age at enrollment: 4.3 [− 0.01, 17.3] y) and 148 (55.0%) were adults (51.4 [18.5, 78.9] y; Table 2). The majority of children and adults were female (61.2 and 73.0%, respectively) and white (57.7 and 90.0%, respectively).

**Table 1:** Number of sites and patients enrolled in the HPP Registry by age at enrollment

	Patients Enrolled,^a^ n			
Country	No. of Sites	Children (age < 18 y)	Adults (age ≥ 18 y)	Total
United States	16	38	76	114
United Kingdom	6	24	13	37
Japan	23	28	2	30
Spain	7	5	18	23
Canada	1	8	13	21
France	2	6	9	15
Australia	2	10	1	11
Russia	1	1	0	1
Italy	3	0	10	10
Germany	3	1	5	6
Portugal	1	0	1	1
Total	65	121	148	269

*HPP* hypophosphatasia

^a^Enrollment dates: January 2015–September 2017

**Table 2:** Demographics by age at enrollment

Characteristic	Children (age < 18 y) (*n* = 121)	Adults (age ≥ 18 y) (*n* = 148)	Total (*N* = 269)
Age at enrollment,^a^ y	*n* = 121	*n* = 148	*n* = 269
Mean (SD)	5.7 (4.7)	48.8 (15.4)	29.4 (24.5)
Median (min, max)	4.3 (−0.01, 17.3)	51.4 (18.5, 78.9)	26.0 (−0.01, 78.9)
Sex, n (%)	*n* = 121	*n* = 148	*n* = 269
Female	74 (61.2)	108 (73.0)	182 (67.7)
Race,^b^ n (%)	*n* = 111	*n* = 140	*n* = 251
White	64 (57.7)	126 (90.0)	190 (75.7)
Asian	35 (31.5)	3 (2.1)	38 (15.1)
American Indian or Alaska Native	0	2 (1.4)	2 (0.8)
Native Hawaiian or other Pacific Islander	0	1 (0.7)	1 (0.4)
Other/multiple	5 (4.5)	4 (2.9)	9 (3.6)
Not reported	7 (6.3)	4 (2.9)	11 (4.4)
Ethnicity,^b^ n (%)	*n* = 121	*n* = 147	*n* = 268
Not Hispanic or Latino	103 (85.1)	116 (78.9)	219 (81.7)
Hispanic or Latino	4 (3.3)	18 (12.2)	22 (8.2)
Not reported	14 (11.6)	13 (8.8)	27 (10.1)
Family history of HPP, n (%)	*n* = 117	*n* = 135	*n* = 252
Yes	50 (42.7)	66 (48.9)	116 (46.0)
No	61 (52.1)	54 (40.0)	115 (45.6)
Not reported	6 (5.1)	15 (11.1)	21 (8.3)
Treated with asfotase alfa at enrollment, n (%)	*n* = 121	*n* = 148	*n* = 269
Yes	45 (37.2)	26 (17.6)	71 (26.4)

*HPP* hypophosphatasia, *SD* standard deviation

^a^Negative values for age indicate enrollment date occurred during pregnancy

^b^The race and ethnicity categories used are those recommended by the US National Institutes of Health

### HPP medical history

**Fig. 1:**
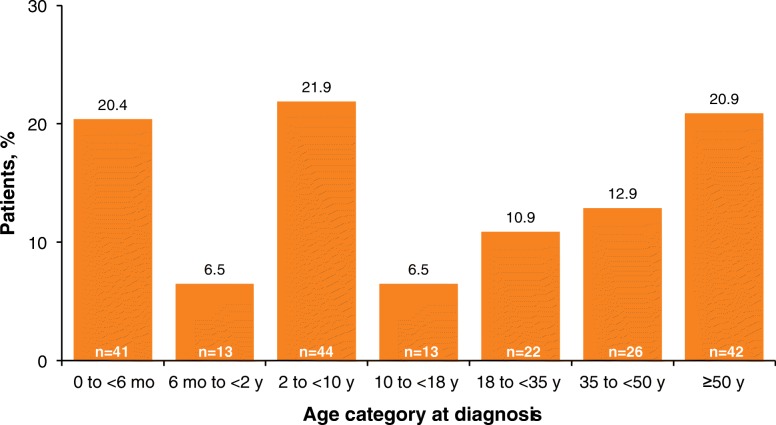
Age category at time of diagnosis of HPP (*n* = 201). *HPP* hypophosphatasia

A family history of HPP was reported for nearly half of the children (42.7%) and adults (48.9%; Table 2). Approximately half of all patients were diagnosed with HPP in childhood (55.2%), and half were diagnosed in adulthood (44.8%). In childhood, diagnoses occurred most frequently between age 2 to < 10 years (21.9%) and before age 6 months (20.4%). Diagnosis during adulthood was most common at age ≥ 50 years (20.9%; Fig. 1).

**Fig. 2:**
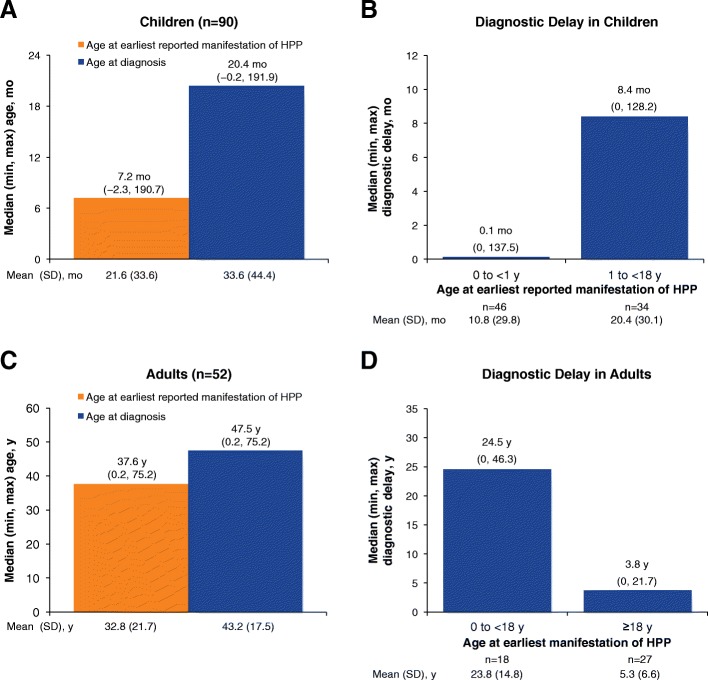
Age at earliest reported manifestation vs. age at diagnosis of HPP (**a**: children; **c**: adults) and diagnostic delay by age at earliest reported manifestation of HPP (**b**: children; **d**: adults). Patients with reported age at first reported manifestation occurring after their age at diagnosis were excluded from the analysis of diagnostic delay. Negative values for age indicate a date that occurred during pregnancy. *HPP* hypophosphatasia

The diagnosis of HPP was delayed relative to age of reported onset of signs or symptoms of the disease in both children and adults (Fig. 2). Children had a median age at earliest reported manifestation of HPP of 7.2 months (min: − 2.3 mo, max: 16.0 y), more than 12 months before median age at HPP diagnosis of 20.4 months (min: − 0.2 mo, max: 16.0 y; Fig. 2 a). Median diagnostic delay was < 1 week (0.1 mo; min: 0, max: 11.5 y) for children who had first reported manifestation of HPP at age < 1 year (*n* = 46) and 8.4 months (min: 0, max: 10.7 y) for children with earliest reported HPP manifestation at age > 1 year (*n* = 34; Fig. 2 b). In adults, median age of earliest reported manifestations of HPP was 37.6 years (min: 0.2 y, max: 75.2 y), which preceded median age at diagnosis (47.5 y; min: 0.2 y, max: 75.2 y) by ~ 10 years (Fig. 2 c). Median diagnostic delay was 24.5 years (min: 0, max: 46.3 y) for adults who had earliest reported manifestations of HPP before age 18 years (*n* = 18) and 3.8 years (min: 0, max: 21.7 y) for those with first reported manifestation at age ≥ 18 years (*n* = 27; Fig. 2 d).

Premature loss of deciduous teeth (48.2% of children aged ≥6 mo), bone deformity (32.5%), and failure to thrive (26.7%) were most commonly reported in the HPP-related disease history of children (Table 3). Pain (74.5%), orthopedic procedures and therapies (44.6%), and recurrent and poorly healing fractures (36.5%) were most commonly reported in the HPP-related disease history of adults (Table 3).

**Table 3:** HPP-related disease history

	n/N (%)	
Category^a^: Symptom/sign	Children (*n* = 121)	Adults (*n* = 148)
Rheumatic	25/119 (21.0)	115/137 (83.8)
Calcific periarthritis	0	9 (6.6)
Chondrocalcinosis	0	6 (4.4)
Fibromyalgia	2 (1.7)	13 (9.5)
Pain^b^	23 (19.3)	102 (74.5)
Pseudogout	2 (1.7)	8 (5.8)
Skeletal	53/120 (44.2)	66/137 (48.2)
Bone deformity	39 (32.5)	18 (13.1)
Pseudofractures	1 (0.8)	9 (6.6)
Recurrent and poorly healing fractures	5 (4.2)	50 (36.5)
Rickets-like changes (by radiograph)	22 (18.3)	8 (5.8)
Orthopedic procedures and therapies	26/120 (21.7)	62/139 (44.6)
Muscular	23/119 (19.3)	51/137 (37.2)
Abnormal gait	17/91^c^ (18.7)	28/137 (20.4)
Weakness	15/119 (12.6)	42/137 (30.7)
Premature loss of deciduous teeth	53/110^d^ (48.2)	34/137 (24.8)
Renal/metabolic	28/119 (23.5)	22/137 (16.1)
Hypercalcemia	15 (12.6)	6 (4.4)
Hyperphosphatemia	7 (5.9)	6 (4.4)
Kidney stones	2 (1.7)	7 (5.1)
Nephrocalcinosis	11 (9.2)	5 (3.6)
Neurologic	32/120 (26.7)	16/137 (11.7)
Craniosynostosis	12 (10.0)	4 (2.9)
Developmental delay	16 (13.3)	3 (2.2)
Increased intracranial pressure	2 (1.7)	1 (0.7)
Seizures	11 (9.2)	12 (8.8)
Failure to thrive	32/120 (26.7)	7/137 (5.1)
Respiratory support	22/118 (18.6)	6/138 (4.3)
Invasive ventilation	18 (15.3)	0
CPAP/BiPAP	7 (5.9)	5 (3.6)
Supplemental oxygen	11 (9.3)	2 (1.4)

*CPAP* continuous positive airway pressure, *BiPAP* bilevel positive airway pressure, *HPP* hypophosphatasia

^a^Patients may have had > 1 sign/symptom within each category

^b^Combines generalized body pain, chronic bone pain, and chronic muscle pain

^c^Excludes patients aged < 2 years at enrollment

^d^Excludes patients aged < 6 months at enrollment

### Medication history

**Fig. 3:**
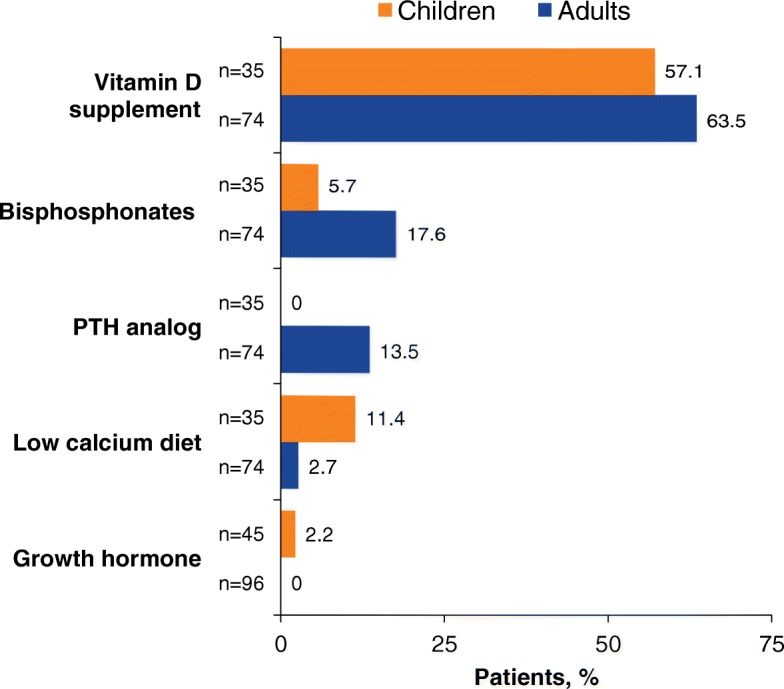
Treatments affecting bone mineralization reported in medical histories for children and adults enrolled in the HPP Registry. *HPP* hypophosphatasia; *PTH* parathyroid hormone

Treatments affecting bone mineralization that were reported in baseline medical histories are summarized in Fig. 3. The majority of children (20/35 [57.1%]) and adults (47/74 [63.5%]) for whom data were available had received vitamin D supplementation. Bisphosphonate use was reported for 2/35 (5.7%) children and for 13/74 (17.6%) adults. Of the total number of medications reported, 14/128 (10.9%) taken by children and 93/143 (65.0%) taken by adults were for pain management and 1/128 (0.8%) taken by children and 35/143 (24.5%) taken by adults were antidepressant or anxiolytic medications. Most patients (73.6%) were not receiving asfotase alfa at enrollment (Table 2).

### *ALPL* pathogenic variants

**Fig. 4:**
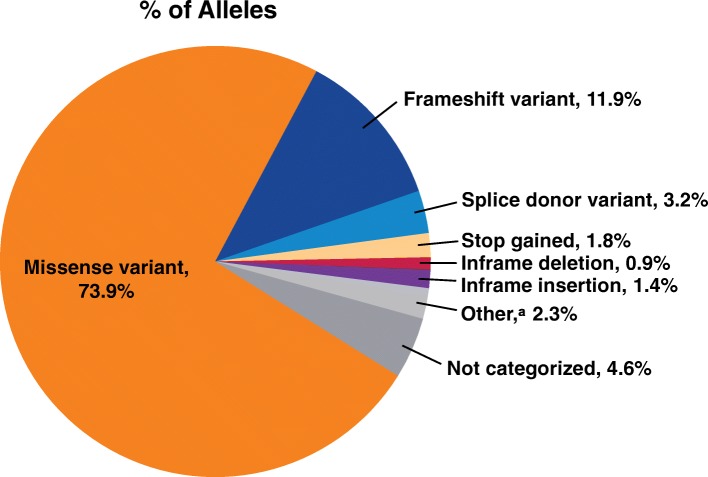
Frequency of *ALPL* pathogenic variants. Pathogenic variants were categorized using the definitions from Sequence Ontology [40]. Figure reports the percentages of the total number of pathogenic variants reported (*n* = 218). ^a^Other includes structural variant (*n* = 2), synonymous variant (*n* = 2), and missense variant/splice region variant (*n* = 1)

In total, 172 (68.8%) of 250 patients with submitted data had *ALPL* pathogenic variant analysis performed. Among the 172 patients (86 children, 86 adults), 218 pathogenic variants (126 in children, 92 in adults) were reported. Missense variants were the most common of all the reported pathogenic variants in children (72.2%) and adults (76.1%) (Fig. 4).

## Discussion

The Global HPP Registry represents, to date, the largest real world dataset from patients with HPP. Here we report baseline characteristics and medical history for up to 269 patients from 11 countries who were enrolled in the Registry during its first 2.75 years of recruitment.

Medical histories showed substantial delays between age at earliest reported manifestations and age at diagnosis of HPP in both children and adults. There may be several reasons for this, including low awareness of HPP, heterogeneity of the manifestations of HPP, and lack of specific or routine testing for HPP. Additionally, as laboratories use different methods for assessment of ALP activity, reference ranges often vary, and it is possible that age- and sex-adjusted ALP reference intervals may not have been used or low values may not have been flagged. The diagnostic delay in the Registry population is concerning, given that nearly half of the patients had a family history of HPP. The index of suspicion for HPP should be high in patients with a positive family history to facilitate a timely diagnosis [3], and our findings suggest that family history is not appropriately considered in the diagnosis.

Diagnostic delays were shorter for children with HPP than for adults, possibly reflecting more obvious clinical manifestations coupled with increased awareness of disease manifestations in recent years, and a more thorough clinical and laboratory evaluation. Prolonged diagnostic delays have been reported previously in adults with HPP [19, 20]. One study of 22 adults with HPP reported a 5-year delay between first signs/symptoms and diagnosis, with the first manifestations of HPP signs/symptoms occurring at a median age of 44 years and diagnosis at a median age of 49 years [19]. A retrospective case review in 9 adults with HPP reported a median diagnostic delay of 46 years since presentation of dental signs of HPP and 27 years since the first fracture or major adult tooth problems [20]. Further, in our Registry study population, certain age groups were diagnosed with HPP more often, with peaks in diagnoses for those aged < 6 months, aged 2 to < 10 years, and aged ≥50 years. This is an interesting observation that may reflect disease severity and/or burden at these ages. Many patients diagnosed with HPP as adults had manifestations in childhood, highlighting the importance of taking thorough medical histories to ensure timely diagnosis.

Baseline medical history from the Registry documented systemic manifestations of HPP across all ages, which were generally consistent with manifestations described in the literature on childhood and adult HPP [19, 21, 22]. Interestingly, skeletal manifestations were present in < 50% of children (44.2%) and adults (48.2%), and specifically, rickets-like changes on radiographs were reported in only 18.3% of children, highlighting the importance of considering nonskeletal manifestations as part of the diagnosis of HPP. Further, recurrent and poorly healing fractures were less common in children (4.2%) than adults (36.5%); however, the data do not reflect whether patients had a history of ≥1 fracture, thereby probably underestimating the overall occurrence of fractures, recurrent fractures, and/or poorly healing fractures.

The need for respiratory support was reported more frequently for children (18.6%) than adults (4.3%). This may be expected because patients with perinatal/infantile HPP often experience respiratory complications, consistent with the natural history of HPP in young children. These data may also be underestimated because patients who were deceased, likely because of respiratory compromise, were not included in the Registry. It is unknown whether the respiratory support reported in the small percentage of adults was provided for symptoms occurring during early childhood or whether this represents respiratory comorbidities.

Pain was commonly reported in the medical histories of patients with HPP (about one fifth of children and three quarters of adults), with approximately 3 times the proportion of adults as children receiving pain medications. The lower rate in children may be an underrepresentation, as young children may be less likely or unable to report pain; it has been reported that parents and medical practitioners often underestimate pain severity in children [23–26]. Our finding in adults is consistent with results of a previous study (*N* = 125), which reported that 95% of adults with HPP had a history of pain and recent pain [22]. Chronic pain contributes to impairments in physical function, ability to perform activities of daily living, and quality of life in patients with HPP [20, 22], as has been shown for other bone diseases (e.g., osteogenesis imperfecta) [27–29].

The Registry also showed that bisphosphonates are being prescribed to patients with HPP (likely before a diagnosis of HPP). Bisphosphonates should be avoided in patients with HPP because of their biochemical similarity to PPi and because they limit bone turnover leading to reduced activity of bone-specific ALP [30–32]. Case studies of adults with previously undiagnosed HPP have reported increased incidences of atypical femoral fractures during treatment with bisphosphonates [33–36].

As with any registry collecting real world data, there are inherent challenges and limitations, such as ensuring complete data entry [37]. Access to and completeness of patient medical records vary across countries because of differences in medical chart documentation practices, limits on time clinicians have to complete data entry, data confidentiality policies, and data linkages within one or more healthcare systems. To address this, missing data were retrospectively collected. Further, data collected for the Registry may have been subject to recall bias, both by the patient and the clinician [37]. In addition, because the Registry did not capture data from patients who were deceased before enrollment, the data may underestimate the full spectrum of disease burden in neonates and young children. Lastly, details on the genotypes of patients in the Registry are not presented, as this information was not available at the time of data analysis [37].

## Conclusions

Baseline data from the HPP Registry provide evidence that delays in the recognition and diagnosis of HPP are common and that HPP is associated with systemic manifestations across all ages. These findings highlight the ongoing need to take thorough medical and family histories and the importance of recognizing the systemic manifestations of HPP to ensure a timely diagnosis. The inclusion of HPP on newborn screening panels should be considered to minimize diagnostic delays and ensure appropriate patient follow-up and management, as has been observed with other genetic diseases [38, 39]. The Global HPP Registry will further improve our understanding of the natural history of HPP and provide longitudinal data on disease burden of HPP, including systemic manifestations, use of concomitant medications that manage manifestations and/or complications of HPP, and potential impact on physical function and quality of life. An improved understanding will allow us to better explore potential geographic, ethnic, and genetic differences in disease manifestations and assess the impact of the approved treatment, asfotase alfa.

## Abbreviations

ALP: Alkaline phosphatase
BiPAP: Bilevel positive airway pressure
CPAP: Continuous positive airway pressure
eCRF: Electronic case report form
HPP: Hypophosphatasia
PLP: Pyridoxal 5′-phosphate
PPi: Inorganic pyrophosphate
PTH: Parathyroid hormone
SD: Standard deviation
TNSALP: Tissue-nonspecific alkaline phosphatase
